# Extraction of high-quality DNA from ethanol-preserved tropical plant tissues

**DOI:** 10.1186/1756-0500-7-268

**Published:** 2014-04-24

**Authors:** Eduardo A Bressan, Mônica L Rossi, Lee TS Gerald, Antonio Figueira

**Affiliations:** 1 Núcleo de Pesquisa em Tecnologia e Inovação para Sustentabilidade da Agricultura, Centro de Energia Nuclear na Agricultura, Universidade de São Paulo, Av. Centenário, 303, CP 96, 13400-970 Piracicaba, SP, Brazil; 2 Centro de Ciências Agrárias, Universidade Federal de São Carlos, Rod. Anhanguera, km 174, 13600-970 Araras, SP, Brazil

**Keywords:** Dehydration, DNA extraction, *Jatropha*, Sample conservation, *Theobroma*, Tissue storage

## Abstract

**Background:**

Proper conservation of plant samples, especially during remote field collection, is essential to assure quality of extracted DNA. Tropical plant species contain considerable amounts of secondary compounds, such as polysaccharides, phenols, and latex, which affect DNA quality during extraction. The suitability of ethanol (96% v/v) as a preservative solution prior to DNA extraction was evaluated using leaves of *Jatropha curcas* and other tropical species.

**Results:**

Total DNA extracted from leaf samples stored in liquid nitrogen or ethanol from *J. curcas* and other tropical species (*Theobroma cacao*, *Coffea arabica*, *Ricinus communis*, *Saccharum* spp., and *Solanum lycopersicon*) was similar in quality, with high-molecular-weight DNA visualized by gel electrophoresis. DNA quality was confirmed by digestion with *Eco*RI or *HindIII* and by amplification of the ribosomal gene internal transcribed spacer region. Leaf tissue of *J. curcas* was analyzed by light and transmission electron microscopy before and after exposure to ethanol. Our results indicate that leaf samples can be successfully preserved in ethanol for long periods (30 days) as a viable method for fixation and conservation of DNA from leaves. The success of this technique is likely due to reduction or inactivation of secondary metabolites that could contaminate or degrade genomic DNA.

**Conclusions:**

Tissue conservation in 96% ethanol represents an attractive low-cost alternative to commonly used methods for preservation of samples for DNA extraction. This technique yields DNA of equivalent quality to that obtained from fresh or frozen tissue.

## Background

Despite technological improvements, conservation of plant tissue samples collected in remote areas for later DNA extraction remains a challenge. Expensive methods are required to maintain the integrity of samples for subsequent extraction of superior-quality DNA. Fresh, dehydrated, or lyophilized tissues are preferred to avoid nucleic acid degradation, but such sample processing is not feasible [1] in certain situations—especially in isolated tropical regions, where significant repositories of biodiversity may occur.

With respect to tropical plant tissues, an additional complication is the presence of secondary compounds, such as phenolics, tannins, latex, and polysaccharides. These compounds hinder the extraction of contaminant-free DNA of sufficient quality for subsequent molecular analyses based on enzymatic digestion, amplification, or next-generation sequencing [2-4].

Many techniques for tissue preservation have been described, such as drying samples at room temperature or in a laboratory oven, preservation on dry ice or in liquid nitrogen, freeze drying, or storage in buffer solutions containing silica gel [5-7]. However, many of the materials required for such methods are not readily available at the collection site. Ethanol, which inactivates enzymes and secondary metabolites, represents a viable alternative for plant tissue preservation [3,8]. In this study, we evaluated the utility of ethanol as an inexpensive preservation solution for plant tissues, especially those from tropical species, for DNA extraction. As part of our investigation, we carried out histological observations to examine the effect of ethanol at the cellular level.

## Methods

### Materials

Recently-expanded leaves of *Jatropha curcas* L., *Theobroma cacao* L. (cacao), *Coffea arabica* L. (coffee), *Ricinus communis* L. (castor bean), *Saccharum* spp. (sugarcane), and *Solanum lycopersicon* L. (tomato) were collected from field- or greenhouse-grown plants. Each leaf sample was divided into two portions: a 2.5-g portion was stored in a 15-mL plastic centrifuge tube containing 8 mL of 96% ethanol for 30 days, while the other half was stored in liquid nitrogen. Fresh samples of *J. curcas* were used for microscopic analyses.

### DNA extraction

For DNA extraction, 50-mg leaf samples from both conservation treatments (ethanol or frozen in liquid nitrogen) were finely ground in liquid nitrogen. The pulverized samples were incubated in buffer (2% cetyltrimethylammonium bromide, 1.4 M NaCl, 100 mM Tris–HCl [pH 8.0], 20 mM EDTA [pH 8.0], 1% polyvinylpyrrolidone [mass weight 10,000], 0.2% β-mercaptoethanol, and 0.1 mg mL^-1^ proteinase K) at 55°C for 60 min [9]. After this step, the solution was extracted twice with chloroform:isoamyl-alcohol (24:1 v/v). DNA was precipitated by the addition of cold isopropanol to the solution followed by centrifugation; the resulting pellet was washed with 70% ethanol and allowed to air dry. The DNA pellet was resuspended in 50 μL TE buffer (10 mM Tris–HCl [pH 8.0] and 0.1 mM EDTA [pH 8.0]) containing ribonuclease A (10 μg mL^-1^) and incubated at 37°C for 30 min [10].

### DNA concentration and quality

DNA concentration was estimated using a DyNA Quant 2000 fluorometer (Amersham Biociences, Buckinghamshire, UK) and a NanoDrop 2000 spectrophotometer (Thermo Scientific, Wilmington, DE, USA). DNA quality was checked by electrophoresis of 50-ng aliquots on a 0.8% agarose gel stained with SYBR Gold (Invitrogen, Eugene, OR, USA).

### DNA digestion

Genomic DNA samples (1 μg) were digested overnight with 10 U of *Eco*RI or *Hin*dIII (Promega, Madison, WI, USA) under recommended conditions at 37°C, and analyzed by electrophoresis on a 0.8% agarose gel stained with SYBR Gold.

### PCR amplification

To evaluate DNA suitability for PCR amplification, primers specific for the internal transcribed spacer (ITS) region of 18S-25S ribosomal DNA (*ITS1-18S*: 5′-CGTAACAAGGTTTCCGTAGG-3′; *ITS4*: 5′-TCCTCCGCTTATTGATATGC-3′) [11] were used for amplification in 20-μL final reaction volumes containing 25 ng DNA, *Taq* polymerase buffer (50 mM KCl, 10 mM Tris–HCl [pH 8.8], and 0.8% Nonidet P40), 1.5 mM MgCl_2_, 100 μM of each dNTP, 0.2 μM of each primer, and 1 U *Taq* polymerase (Fermentas Life Sciences, Burlington, Canada). Amplifications were conducted as follows: initial denaturation at 94°C for 3 min, followed by 35 cycles of 30 s at 94°C, 1 min at 58°C, and 1 min at 72°C, with a final extension at 72°C for 7 min. Amplification products were analyzed by electrophoresis on a 1.5% agarose gel stained with SYBR Gold.

### Light microscopy (LM) and transmission electron microscopy (TEM)

We analyzed *J. curcas* leaf samples stored in 96% ethanol for either 1 h or 30 days, with freshly collected leaves used as a control. The samples were fixed for 48 h in a solution of 0.05 M sodium cacodylate buffer (pH 7.2) containing 2% glutaraldehyde, 2% paraformaldehyde, and 5 mM CaCl_2_[12]. The samples were then washed in 0.1 M sodium cacodylate buffer and fixed for 1 h at room temperature with 1% osmium tetroxide in the same buffer. Dehydration was performed in an increasing series of acetone in water (30–100%), with the samples subsequently infiltrated and embedded in Spurr resin for 48 h. Semi-thin sections (120–200 nm) were collected on glass slides, stained with 2% toluidine blue in water for 5 min, rinsed in distilled water, and air dried. The sections were permanently mounted in Entellan resin and observed and documented under a light microscope (Axioscop 2; Zeiss, Jena, Germany). Ultrathin sections (60–90 nm) were collected on copper grids (300 mesh) and stained with 2.5% uranyl acetate followed by lead citrate [13]. The sections were observed at 80 kV using a transmission electron microscope (Zeiss EM 900).

## Results

**Figure 1:**
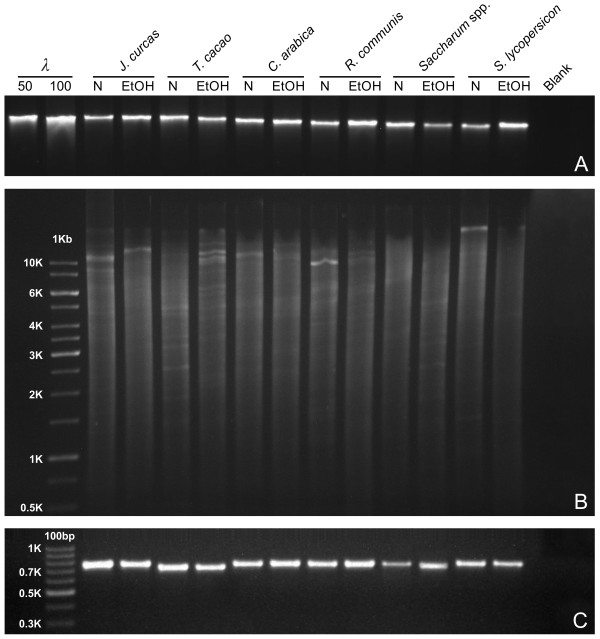
**Comparative analysis of DNA from leaf samples conserved in ethanol vs. liquid-nitrogen-frozen controls. (A)** λ DNA (50 and 100 ng) followed by non-digested genomic DNA (50 ng) from tropical plant species *Jatropha curcas*, *Theobroma cacao*, *Coffea arabica*, *Ricinis communis*, *Saccharum* spp., and *Solanum lycopersicon* analyzed by 0.8% agarose gel electrophoresis. **(B)** 1-Kb DNA mass ladder (Fermentas) and 1 μg *Eco*RI-digested DNA from *J. curcas*, *T. cacao*, *C. arabica*, *R. communis*, *Saccharum* spp., and *Solanum lycopersicon*. N – liquid nitrogen and EtOH – ethanol. **(C)** 100-bp DNA ladder molecular weight marker (Fermentas) and ITS PCR amplification products of the same plant species.

Leaf samples stored prior to extraction in liquid nitrogen or 96% (v/v) ethanol from *J. curcas*, cacao, coffee, castor bean, sugarcane, and tomato yielded DNA of similar quality. High-molecular-weight DNA without signs of degradation was detected by gel electrophoresis (Figure 1A). DNA yields were in the range of 2.3–6.2 μg g^-1^ tissue fresh weight (FW), with frozen samples giving a higher yield (4.1–6.2 μg g^-1^ tissue FW). Samples conserved in ethanol produced similar yields among species: 2.3 μg g^-1^ for *J. curcas*, 3.1 μg g^-1^ for cacao, 2.7 μg g^-1^ for coffee, 2.6 μg g^-1^ for sugarcane, 3.4 μg g^-1^ for castor bean, and 2.9 μg g^-1^ for tomato. Preservation in ethanol appeared to minimize contaminants and produced good-quality DNA with OD_260_/OD_280_ values in the range of 1.83–1.97. All samples were amenable to successful digestion by *Eco*RI (Figure 1B) or *Hin*dIII (not shown) under the tested conditions. ITS amplification products of expected sizes were successfully generated from all samples (*J. curcas*: ~755 bp, cacao: 774 bp, coffee: 703 bp, castor bean: ~740 bp, sugarcane: ~680 bp, and tomato: 697 bp) (Figure 1C).

**Figure 2:**
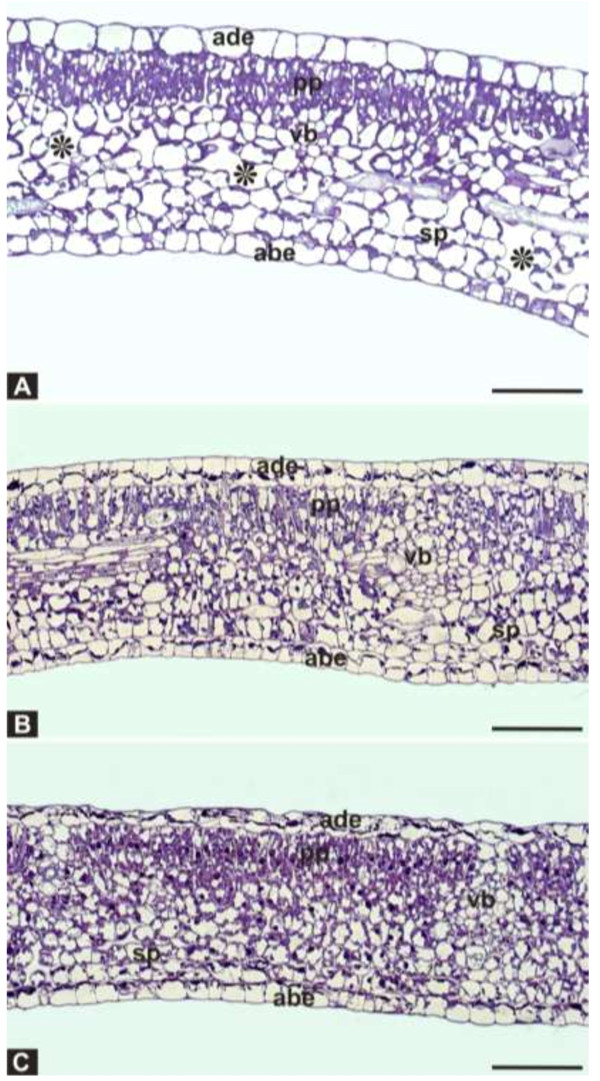
**Effect of ethanol conservation treatment on leaf cross sections analyzed by light microscopy (LM).** Cross section showing general view of dehydrated cells of *J. curcas* leaves observed by LM. **(A)** Control. **(B)** 1 h in ethanol. **(C)** After 30 days in ethanol. Abbreviations are as follows: adaxial epidermis (ade), palisade parenchyma (pp), vascular bundle (vb), spongy parenchyma (sp), and abaxial epidermis (abe). *intercellular space. Bar: LM - 50 μm.

**Figure 3:**
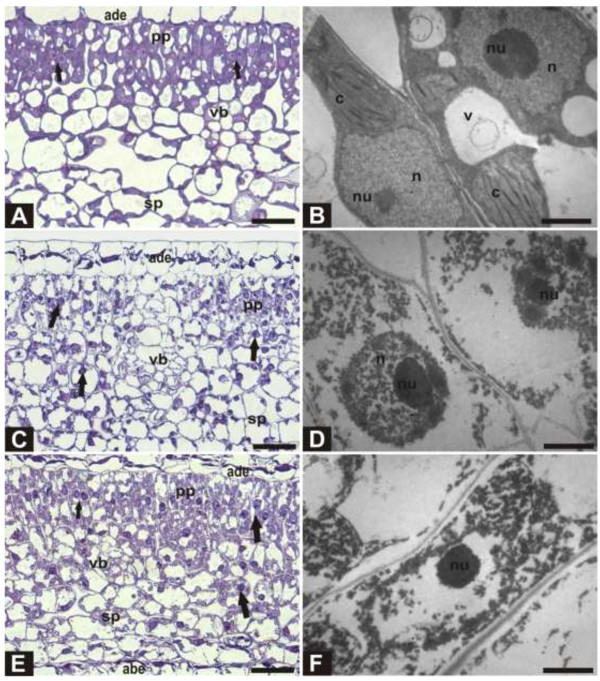
**Microscopic analysis of leaf samples conserved in ethanol compared with fresh leaf controls.** Cross section of *J. curcas* leaves observed by light (LM) and transmission electron (TEM) microscopy. **(A)** Control (LM). **(B)** Control (TEM). **(C)** 1 h in ethanol (LM). **(D)** 1 h in ethanol (TEM). **(E)** After 30 days in ethanol (LM). **(F)** After 30 days in ethanol (TEM). Abbreviations are as follows: adaxial epidermis (ade), palisade parenchyma (pp), vascular bundle (vb), spongy parenchyma (sp), abaxial epidermis (abe), nucleus (n), nucleolus (nu), chloroplast (c), and vacuole (v). Arrows: genomic DNA; Bar: LM - 50 μm; TEM - 2 μm.

Finally, fresh and ethanol-stored leaf samples of *J. curcas* were analyzed by LM and TEM. Soaking the tissues in ethanol caused cell dehydration and cell shrinkage, with an important decrease in cell volume (Figures 2A–C; 3A, C, E). Histological analysis under LM revealed important anatomical alterations caused by the ethanol treatment (Figure 3C, E) in comparison with fresh tissues (Figure 3A). Under ethanol treatment, most nuclei from leaves of *J. curcas* appeared to be well preserved with intensely stained nucleoli, suggesting the precipitation of nucleic acids (Figure 3A, C, E: arrows). Most of the other cellular constituents were leached from the cells.

The changes observed under ethanol treatment were confirmed by TEM. Treatment with ethanol cleared cellular contents, while the nuclear membrane and other components—including the nucleolus—were apparently maintained (Figure 3B, D). Nucleic acids appeared to be contained in cellular compartments. After 30 days in ethanol, cell contents were removed to a large extent with the disintegration of the cytoplasm (Figure 3F). Occasional short fragments were still observed inside the nucleus near the nucleolus. Presumed condensed regions of chromatin of isolated nuclei were prominent and remained adherent to the nucleoli. Some free fragments of chromatin were also observed (Figure 3F).

## Discussion

Successful extraction of nucleic acids from tissues preserved in ethanol has been previously demonstrated [8]. Soaking tissues in ethanol appears to facilitate tissue lysis, cell wall disruption, and deactivation of DNAases [1,14,15]. Previous studies have indicated that short (30–60 min) pretreatment of plant tissues in ethanol or other organic solvents improves DNA quality [14]. Conversely, Pyle and Adams [16] found that preservation of spinach leaves in 95% ethanol for as little as 24 h resulted in significant DNA degradation.

In this study, we determined that DNA can be successfully extracted from leaf tissue samples of tropical species preserved in ethanol for long periods (over 30 days). A viable alternative to other methods for fixation and conservation of DNA, ethanol preservation may reduce or inactivate secondary metabolites that can contaminate or degrade genomic DNA [3,8]. It is noteworthy that the cell walls of *J. curcas* were partly disrupted when exposed to ethanol for 30 days, facilitating subsequent genomic DNA extraction. Similar results have been uncovered in yeast (*Saccharomyces cerevisiae*), *Arabidopsis thaliana*, and *Daucus carota*[15].

The fundamental structure of primary cell walls of all land plants appears to be similar: cellulose microfibrils embedded in a hydrated matrix composed mostly of neutral and acidic polysaccharides and small amounts of structural proteins [17]. Treatment of plant tissues with ethanol triggers a series of cellular chemical events, which leads to protein denaturation, matrix dehydration, cellular metabolism disruption, and precipitation of nucleic acids with more than 15 nucleotides. At the same time, the development of opportunistic microorganisms in samples is inhibited [18,19].

Treatment with ethanol softened the tissues for DNA extraction. The mode of action of ethanol in the cell walls was not apparent by microscopy; however, protein denaturation and polysaccharide matrix dehydration may favor the displacement of cellular aqueous components by ethanol, leading to cell membrane disruption and consequent reduction or inactivation of secondary metabolites that can contaminate or degrade DNA [18].

## Conclusion

Tissue conservation in 96% ethanol represents an attractive low-cost alternative to other methods used for preservation and transport of samples for DNA extraction. This technique is especially valuable for field collection from remote regions or during low budget initiatives, and yields DNA of equivalent quality to that obtained from fresh or frozen tissue.

## Competing interests

All authors certify that there is no conflict of interest with any financial organization regarding the material discussed in the manuscript.

## Authors’ contributions

EAB designed the experiments, carried out the laboratory work, and drafted the manuscript. AF and LTSG participated in the study design and wrote the manuscript. MLR carried out the microscopic studies. All authors participated in writing and revising the manuscript and approved the final version.
